# Metamizole versus placebo for panretinal photocoagulation pain control: a prospective double-masked randomized controlled study

**DOI:** 10.1186/s40942-015-0021-8

**Published:** 2015-11-12

**Authors:** Rafael Barbosa de Araújo, Leandro Cabral Zacharias, Breno Marques de Azevedo, Beatrice Schmidt Giusti, Rony Carlos Pretti, Walter Y. Takahashi, Mário Luiz Ribeiro Monteiro

**Affiliations:** 1 Av. Dr. Enéas de Carvalho Aguiar, 255 Cerqueira César, São Paulo, 05403-000 Brazil; 2 Hospital das Clínicas of University of Sao Paulo Medical School-HCFMUSP, São Paulo, Brazil

**Keywords:** Diabetic retinopathy, Panretinal photocoagulation, Metamizole, Analgesia, Pain score

## Abstract

**Background:**

Diabetic retinopathy is one of most common and threatening ocular diseases. Many of these patients need to be submitted to panretinal photocoagulation (PRP), experiencing a significant level of pain. The purpose of this study is to evaluate the effectiveness of oral metamizole in reducing pain during PRP in patients with proliferative diabetic retinopathy (PDR) and very severe non-proliferative diabetic retinopathy (VSNDR).

**Methods:**

Patients from a single center with PDR or VSNDR and indication of bilateral PRP were recruited for a double-masked, controlled, prospective study. The treated eyes were randomly assigned in two groups, and each patient had one eye assigned per group. Group A received 1000 mg of metamizole and group B received a placebo pill 40 min before the laser treatment. The groups were switched for the treatment of the fellow eye. Each patient scored the pain sensation immediately after each PRP section using Scott’s visual analogue scale (VAS). The paired Student t test was used to measure the significance between the two groups VAS scores, with significance level adopted of *p* < 0.05.

**Results:**

Twenty-one patients were recruited. The level of pain was significantly lower when submitted to PRP after oral metamizole treatment compared to placebo (p = 0.002). The mean pain scores for groups A and B were 4.72 ± 1.708 and 5.89 ± 1.967, respectively. The minimum/maximum scores within groups A and B were 1/8 and 1/10, respectively.

**Conclusions:**

The use of 1000 mg of metamizole 40 min before PRP significantly reduces the pain associated with the procedure in patients with PDR or VSNDR.

## Background

Diabetic retinopathy is one of the most important causes of visual impairment in adult population. The standard treatment in the last few decades for eyes with high risk of visual loss, with very severe nonproliferative diabetic retinopathy (VSNDR) or proliferative diabetic retinopathy (PDR), has been the administration of laser panretinal photocoagulation (PRP) [1–4]. The Diabetic Retinopathy Study and, subsequently, the Early Treatment Diabetic Retinopathy Study demonstrated the efficacy of photocoagulation treatment for these situations [5].

Most of the patients undergoing PRP treatment complain about moderate to severe pain sensation. Previous studies show that 73 % of the patients submitted to PRP report painful sensation during the laser treatment [6–8]. Different strategies have been tried to relieve the level of pain associated with the procedure, such as peribulbar anesthesia [6, 9, 10], oral [11] and topical diclofenac [12], oral diazepam, oral mephenamic acid, oral acetaminophen or intramuscular ketorolac tromethamine [6], but there is no solid consensus or good evidence of the efficacy for any of them [13].

Metamizole, also known as dipyrone, is widely used as an analgesic in many countries (Switzerland, Germany, France, Spain, Latin and South America, Far East and Africa) [14]. Although its mechanism of action is not well known, inhibition of cyclooxygenase has been demonstrated, with a peak of action 40 min after the administration [14–16]. Despite the controversial increased risk of metamizole-associated hematological adverse reactions, several studies have used this drug as a comparative analgesic in post-operative analgesy [17–21], and eye surgery trials [22], with well documented safety characteristics by several papers [14, 23–25].

The purpose of our study is to analyze if metamizole, a common oral analgesic widely used in South America, is effective in reducing painful sensation during retinal laser treatment in patients with PDR or VSNDR [6, 11].

## Methods

We performed a randomized, double-masked clinical trial to evaluate the efficacy of metamizole as a pre-emptive analgesic agent during PRP. Patients with bilateral PDR or VSNRD from the Ophthalmology Service of the Hospital das Clínicas—University of São Paulo Medical School that were recruited over a 2-year period were included. All patients signed an informed consent form and the ethics committee approval was obtained before the beginning of the study.

Inclusion criteria were: bilateral proliferative diabetic retinopathy or very severe non-proliferative diabetic retinopathy, no previous laser treatment, best corrected visual acuity of 20/200 or better, intraocular pressure under 21 mmHg, spherical equivalent of ±5.00 diopters, clear media and vitreous, diabetes diagnosed after 30 years of age. The exclusion criteria were: previous photocoagulation treatment, media opacity such as cataracts, corneal diseases or vitreous hemorrhage, unilateral PDR, chronic use of analgesics or history of any side-effects related to metamizole use.

All patients were submitted to PRP, divided in two sessions per eye. PRP was performed by two specific authors (RBA and BMSA) who were blind to the pre-emptive treatment in all sessions. A third professional (BSG) was responsible for administering the drug or the placebo to the patient. The eyes of each patient were randomized into groups A and B. Randomization tables were sent to the pharmacist who allocated each pill in a randomized sequence. Group A eyes received 1000 mg of metamizole 40 min before the laser session and group B eyes were medicated with a placebo pill. Each patient had one eye on group A and one eye on group B. Therefore, a group of patients took metamizole before any PRP, and had PRP with placebo at the fellow eye, while a group of patients started PRP with placebo pills, and had PRP with metamizole when treating the second eye.

Before the laser treatment sessions, each patient was submitted to a complete ophthalmological examination, that consisted of best-corrected visual acuity (BCVA) measurement, biomicroscopy, gonioscopy, tonometry and fundoscopy. Each patient had his or her pupils dilated with topical 1 % tropicamide. The laser treatment was performed with green argon double-frequency laser with a panretinal contact lens. Each session consisted in approximately 500 spots, with laser energy adjusted to achieve moderate white burns, spots size of 250 micrometers and exposure time of 0.2 s. The inferior and nasal retina was treated in the first session, and superior and temporal retina in the second session.

The painful sensation scores were evaluated immediately after finishing each session with the Scott’s Visual Analogue Scale (VAS), that ranges from 0 (no pain at all) to 10 (the worst pain imaginable).

Statistical analysis was expressed in mean, maximum, minimum scores obtained, and standard deviation. Sample size was calculated considering a pain score difference of 2 points between both groups, with Alfa error of 5 % and beta error of 20 %. All data was processed with SPSS version 20.0. The paired Student t test was used to measure the significance between the two groups, with significance level adopted of *p* < 0.05.

## Results

A total of 21 patients (42 eyes) were recruited and their characteristics are summarized in Table 1. The eyes of each patient were randomized to groups A and B. There was no statistically significant difference in gender, age, race and diagnosis (type I or II diabetes) between groups. The mean age of the patients was 52.90 ± 9.42 years, ranging from 31 to 66 years.

**Table 1:** Demographic data from both treated groups

Group characteristics		
Age		
Mean value (years)		52.9 (±9.4)
	N	%
Sex		
Female	10	47.6
Male	11	52.4
Education		
Preliminary school	11	52.4
High school	8	38.1
None	2	9.5
Race		
White	12	57.1
Black	4	19.0
Miscegenous	5	23.8
Diabetes		
DM type 1	4	19.0
DM type 2	17	81.0
Total	21	100.0

All patients took their appropriate pretreatment medications (metamizole or placebo, for groups A or B respectively), underwent their first PRP session, and answered the pain questionnaire. At the very next visit (1 week later), patients were submitted to the same PRP procedure at the fellow eye and therefore took a different pill (Placebo for those that had taken metamizole; metamizole for the patients that had taken placebo).

Laser parameters delivered to the patients were similar between the groups and there was no statistical difference between groups regarding the number of spots delivered, as shown in Table 2. Pain score differences based on the retinal area treated.

**Table 2:** Laser treatment specifications of both group A and B: number of spots per session

Number of spots	Minimum	Maximum	Mean	SD
Group A	424	603	509.33	42.89
Group B	266	576	496.90	58.85

There was no statistical difference between both groups A and B concerning the average number of laser spots per session

The mean VAS pain scores for groups A and B were 4.72 ± 1.71 and 5.89 ± 1.97 (p < 0.01), respectively. The minimum/maximum scores within groups A and B were 1/8 and 1/10, respectively (Table 3). There were no significant differences in the pain scores between the groups regarding gender, race, age or educational level, or to laser intensity parameters. The laser parameters values are shown in Table 4.

**Table 3:** Visual analogue scale pain scores, with mean and standard deviation (SD), maximum and minimum values for each group

Treatment groups	Metamizole		Placebo		ρ value
	Mean ± SD	(Minimum–maximum)	Mean ± SD	(Minimum–maximum)	
VAS (visual analogue scale)	4.72 ± 1.71	(1–8)	5.89 ± 1.97	(1–10)	0.002

The mean pain score value was higher in the placebo group, in comparison with the metamizole group. Statistical significance is showed on the table. Note that only in the placebo group the maximum pain score was achieved

**Table 4:** Laser treatment parameters between groups A and B

	Number of spots		Aim size (microns)		Power (milliwatts)		Exposure time (milliseconds)	
	Placebo	Metamizole	Placebo	Metamizole	Placebo	Metamizole	Placebo	Metamizole
Mean	496.90	509.33	245.24	245.24	277.62	276.90	200.00	200.00
SD	58.85	42.88	14.85	14.85	87.08	88.02	0.00	0.00
*p* value	0.32		1.00		0.31		1.00	

No difference was found between groups A and B regarding laser parameters

Patients referred a significant lower level of pain during PRP when submitted to previous oral metamizole treatment when compared to placebo (p = 0.002). No serious adverse events or drug allergy related to metamizole were reported.

## Discussion

Most patients undergoing PRP typically experience pain during the procedure, which may be so uncomfortable that there is a risk of inadequate treatment being applied or perhaps the patient may even default from attendance [7].

There are numerous options available to reduce or prevent it. Retrobulbar, peribulbar, or subtenon anesthesia are effective pain-relieving procedures, but are invasive for patients and possess risk of potential complications and therefore are not feasible for in-office daily routine [16, 26]. On the other hand, previous results [6] suggest that oral diazepam, acetaminophen, mefenamic acid, and intramuscular injection of ketorolac tromethamine are not effective to reduce pain severity associated with PRP. One study found reasonable results in reducing pain scores with oral etoricoxib [27].

This study sought to find an inexpensive, safe and easy-to-administer method to reduce the pain intensity felt during PRP. It would make the treatment more comfortable for patients, would likely decrease the need for rescue injection anesthesia, and could potentially improve compliance with follow-up PRP treatments [11].

Metamizole is a non-opioid analgesic and one of the most frequently used analgesics around the world, therefore it was chosen for this study to evaluate its analgesic effects. However, in some countries, the drug has been avoided since the 1970s because of what was thought to be an unacceptable risk of agranulocytosis. Recent scientific data do not justify this reasoning [16].

Its mechanism of action is still under discussion, but the main pathway is attributed to an inhibition of prostaglandin synthesis in both peripheral tissues and the central nervous system [17].

The results of this study show a positive effect of metamizole in reducing pain caused by PRP. It is known that pain perception is a personal experience to each individual. Many factors can influence this perception, including gender, cultural differences, past experiences, and anxiety levels [17].

In order to avoid any kind of bias, we selected bilateral cases that had never experienced laser before. Moreover, the same patient was submitted to PRP after either placebo or metamizole (only the order of it was randomized). Our intention was to create the best scenario in order to make unbiased comparison.

The main limitations of our study are the lack of assessment of differences regarding the pain report between first and second laser session or between different retinal areas, which should be aim of further studies. Despite the significant results, this is a small trial and our results should be confirmed by other future studies.

Furthermore, an important observation in this study was the similar laser parameters applied to bot. The occurrence of pain in PRP may be influenced by many parameters, such as duration, intensity, spot size and obviously the number of spots delivered in each session. Recent literature evaluates the pain response with reduced exposure laser times and micropulse technology, with good pain score outcomes [12, 18, 19]. The parameters used during our study were carefully selected before its start. However, patients experiencing severe pain during PRP may ask to stop treatment. Fortunately, our analysis showed that there was no difference in number of spots delivered among groups; therefore we believe this kind of bias did not interfere with our outcomes.

## Conclusion

In conclusion, PRP is a painful treatment for most patients. According to our study, the use of 1000 mg of metamizole 40 min before PRP significantly reduces the pain associated with the procedure in patients with proliferative diabetic retinopathy or VSNDR. Therefore, metamizole is a safe and cheap option that can be applied before the procedure, especially in patients with no history of drug allergy, resulting in more comfort to patients with PDR or VSNDR.

## Abbreviations

PRP: panretinal photocoagulation
PDR: proliferative diabetic retinopathy
VSNDR: very severe non-proliferative diabetic retinopathy
VAS: visual analogue scale
BCVA: best corrected visual acuity
DM: diabetes mellitus
SD: standard deviation
